# A Meta-Analysis of Social Skills Interventions for Preschoolers with or at Risk of Early Emotional and Behavioral Problems

**DOI:** 10.3390/bs13110940

**Published:** 2023-11-16

**Authors:** Xin Dong, Mack D. Burke, Gilbert Ramirez, Zhihong Xu, Lisa Bowman-Perrott

**Affiliations:** 1 Department of Teacher Education, Nicholls State University, Thibodaux, LA 70301, USA; 2 School of Education, Baylor University, Waco, TX 76706, USA; mack_burke@baylor.edu; 3 Department of Global Health, Florida International University, Miami, FL 33199, USA; girami@fiu.edu,; 4 Department of Agricultural Leadership, Education, and Communications, Texas A&M University, College Station, TX 77843, USA; zhihong.xu@ag.tamu.edu; 5 Department of Educational Psychology, Texas A&M University, College Station, TX 77843, USA; lbperrott@tamu.edu

**Keywords:** social skills interventions, preschoolers, emotional and behavioral problems, meta-analysis

## Abstract

Early social–behavioral intervention that emphasizes social skill training is critical to addressing emotional and behavioral problems in early childhood. In this meta-analysis review, we examined all the social skills intervention studies for preschoolers with, or at risk of, emotional and behavioral problems using group designs. This review included 25 studies that met the inclusion criteria. The robust variance estimation method was used to calculate the overall effect size of all the included studies, as this method can count for the pre-existing difference between the experiment and control groups. The included studies yielded an overall effect of 0.54 from the 151 effect sizes that were obtained for the 3484 preschool participants. Curriculum, integration, and treatment fidelity were identified as significant moderators of effects.

## 1. Introduction

An increasing number of children in early childhood settings exhibit problem behaviors and lack adequate social, emotional, and behavioral skills [1]. Research suggests that between 10% and 20% of preschool children exhibit some form of emotional and behavioral difficulties (e.g., [2,3]). Young children with behavior problems are often ignored or rejected by their peers, have adjustment problems upon entering school [4,5], and have problems learning and performing age-appropriate skills [6]. However, these children usually are not formally identified as having emotional and behavioral disorders or provided with special education services at such a young age [7].

Early intervention in the social–behavioral domain is critical for children with or at risk of emotional and behavioral problems. It is critical to teach young children social skills, such as how to establish and maintain relationships with peers and adults, recognize and manage their emotions, follow directions, and request help in the classroom. These social skills can help the children meet behavioral expectations in class and are beneficial for their academic study.

On the other hand, a developmental trajectory of behavior problems in early childhood often puts children on a path towards poor long-term outcomes without effective interventions based on longitudinal studies [8]. Without intervention, many of the preschoolers with conduct problems will start a trajectory that leads to more serious emotional, behavioral, and related mental health problems [9]. One-third of the children with early externalizing behavior problems continue on a trajectory of problem behavior through adolescence and adulthood [10]. Along the same line, Sentse and his colleagues [11] found that children with early onset conduct problems are at heightened risk for internalizing, externalizing, and social adjustment problems. Social skills interventions (SSI) are one common approach to remediate children’s social skills deficits, reduce problem behaviors, and prevent further problems in their lives.

### 1.1. Social Skills

Children with early emotional and behavioral problems often have social skills deficits that lead to challenging behaviors in classroom settings. There is no congruent definition for social skills in the field. Depending on the theoretical orientation, multiple definitions have been used. Merrell and Gimpel [12] indicated that more than 16 definitions of social skills exist within the research literature. One definition that is often used was put forward by Gresham [13], who defined social skills as “behaviors that, within given situations, predict important social outcomes for children” (p. 7). Thus, social skills are highly correlated with children’s social outcomes. Children with adequate social skills are more likely to establish and maintain good relationships with teachers and peers, succeed academically, and feel connected to school [14].

Moreover, according to Gresham’s conceptual framework for social skills training, social skills can be categorized into three domains: (1) social interaction, (2) prosocial behavior, and (3) social–cognitive skills [14,15]. *Social interaction* is defined as the social exchange between two individuals, and it encompasses a variety of skills and behaviors. For example, social initiation skills and turn-taking skills are critical skills for preschoolers to develop relationships. *Prosocial behaviors* refer to a range of voluntary actions directed at other people’s benefit [16]. For example, sharing is one of the earliest prosocial behaviors for children. *Social–cognitive skills* refer to the ability to recognize and regulate emotions and the ability to define problems and generate and consider alternative solutions. For example, interpersonal problem-solving [17] and emotional self-regulation skills [18] are social–cognitive skills.

### 1.2. Social Skills Interventions

Social skills interventions are an important approach in the remediation of children’s problem behaviors and the prevention of further social–behavioral problems [19]. Gresham, Cook, Crews, and Kern [15] broadly defined social skills interventions as “behavioral, cognitive, or social interventions that were directed at training specific skills and/or remediating particular social skills deficits” (p. 33). In early childhood, social skills development mainly focuses on skills related to social interaction with peers, including play skills, social communication skills, socio-emotional skills, and friendship skills. Thus, social skills interventions mainly aim at improving children’s play skills, social communication skills, and social–emotional skills.

### 1.3. Previous Research

In reviewing the literature on SSIs, most of the previous meta-analyses included a wider age range of children. There was no meta-analysis that only focused on preschoolers who are at risk of emotional and behavioral problems, so these meta-analyses could provide limited inferences for the practice in preschool settings. Moreover, they have inconsistent findings regarding the moderating effect of age on the intervention’s impact on social skills. Among the meta-analyses that include preschooler participants, some reported higher effect sizes for preschoolers compared to older children. For example, Schneider and Byrne [20] conducted a meta-analysis on social skills training for children aged 3–19 years old. The effect sizes measured by the mean differences between the experimental and control groups for preschoolers (*M* = 0.97) were more effective than those of elementary school children (*M* = 0.49) and adolescents (*M* = 0.87). They also found training was more effective for withdrawn children (*M* = 1.04) than for aggressive children (*M* = 0.69). In another review, Beelmann, Pfingsten, and Lösel [21] reviewed 49 studies on social competence training for children aged 3–15 years and found an effect size of 0.47. The mean effect size for preschoolers (*M* = 0.96) was much bigger than those of elementary school students (*M* = 0.33–0.35) and adolescents (*M* = 0.45). Moreover, they found greater effects for specific social skills areas (*d* ranging from 0.34 to 0.77) than for broader constructs (e.g., social adjustment, *d* ranging from 0.06 to 0.18). It is quite intuitive as changes in specific social skills areas may be easier to detect, but it may be more challenging to detect changes in broader constructs like social competence. Lastly, Schneider [22] conducted a quantitative review of 79 studies on social skills training to enhance peer relationships for children aged 3–17 years. The overall short-term effect size was *r* = 0.40 (equivalent to *d* = 0.89), suggesting a moderate effect. As to the moderating effect of age, they found a bigger effect size for younger children, but the difference was not significant. With participant diagnosis as the most powerful predictor of effect size in the multivariate analysis, withdrawn children (Fisher *z* = 0.69) responded better than aggressive children (*z* = 0.37) and unpopular children (*z* = 0.37). The dependent variable of focus in this review was peer relationship, for which it may not be easy to find a valid measurement.

However, some researchers found smaller effect sizes for younger children’s behaviors. For example, Lösel and Beelmann [10] conducted a meta-analytic review of 84 research reports for social skills training programs for children aged 0–18 years old. The mean post-intervention effect was *d* = 0.38, whereas the mean follow-up effect size was *d* = 0.28. The mean post-intervention effect size for preschoolers (4–6 years) (*d* = 0.31) was smaller than for elementary students (*d* = 0.39) and adolescents (*d* = 0.41). This finding is controversial to the findings of other reviews, which also bring up the need for an updated review.

Collectively, the previous quantitative reviews have indicated mixed results from the SSI literature. The overall effect of SSIs tends to be moderate in the short term with negligible long-term effects. Thus, it calls for an updated meta-analysis on social skills interventions for preschoolers with emotional and behavioral problems because children’s social skills are developmental and children in different age groups have different needs. Furthermore, the results from the previous meta-analysis are not very informative to practitioners in preschool settings.

### 1.4. Purpose and Research Questions

Previous reviews did not focus exclusively on preschoolers and tended to include young children with other age groups. Evidence shows that the effect size of social skills interventions differs for children with different diagnoses in Schneider’s review [20]. As the purpose of the present review was to specifically examine the effect of the group social skills interventions on preschoolers with emotional and behavioral problems in preschool settings, the following research questions were posed:

1. What is the overall effect of SSIs for preschoolers identified with or at risk of early emotional and/or behavioral problems?
2. What are the effect sizes of SSIs on outcomes related to (1) social–cognitive skills, (2) problem behaviors, and (3) social competence?
3. Are there differential effects for SSIs based on intervention tier, integration, duration, literacy component, curriculum, and treatment fidelity?

Based on the findings from the previous reviews, the authors hypothesized the overall effect of SSIs for preschoolers with emotional and behavioral problems would be medium. The effect sizes of SSIs on outcomes related to (1) social–cognitive skills and (2) problem behaviors would be bigger than the effect size on social competence as social competence is a broader construct compared to the other two types of dependent variables. The potential moderators listed in research question 3 may show a difference in the effect sizes.

## 2. Method

### 2.1. Literature Search

**Figure 1:**
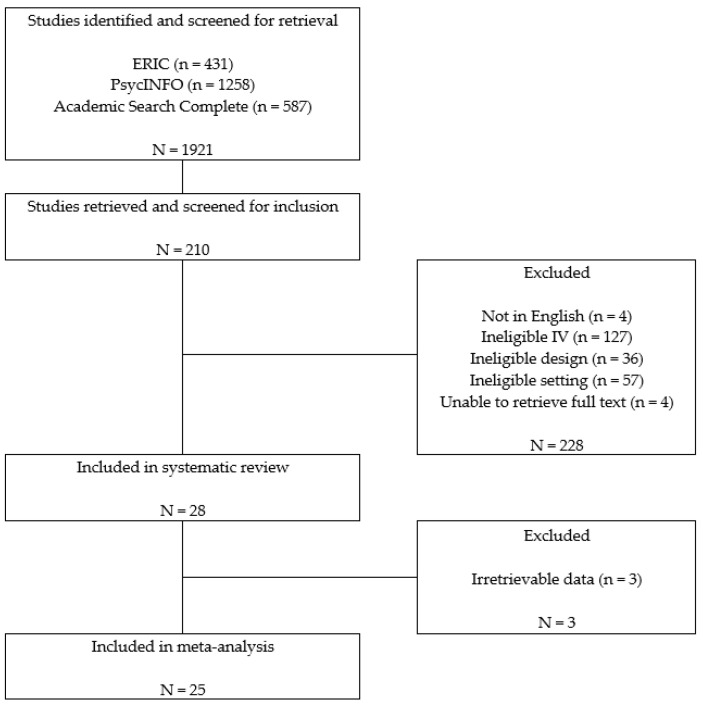
Results of the literature search and inclusion screening.

Figure 1 provides an overview of the search procedures. The search covered studies published from 1970 to November 2017. Databases searched were ERIC, PsycINFO, and Academic Search Complete. Multiple combinations of the following search terms were used: interpersonal competence, prosocial behavior, social cognition, emotional development, social and emotional development, social skills, socio-emotional, social competence, social interaction; disab*, handicapped, emotional disturbances, seriously emotional disturbance, emotional behavioral disorder, EBD, emotional disturbance, emotional problems, behavioral problems, developmental disabilities, developmental delays, learning disabilities, impairment. For Academic Search Complete, the following search terms were applied to limit the participants’ age ranges: preschoolers, 3–5 years, early childhood education, young children, and preschool. For ERIC and PsycINFO, no age limit was applied as there is no place to put the age limit in these databases.

### 2.2. Inclusion and Exclusion Criteria

Eight inclusion criteria were applied to the search results and are listed as follows.

1. Peer-reviewed journals: Studies had to be published in peer-reviewed journals to ensure the quality of the included studies was relatively high. Dissertations, presentation reports, or studies not published in peer-reviewed journals were excluded. Studies not written in English were also excluded.
2. Participants: Studies had to include preschoolers or young children aged 3–6 years old identified as with or at risk of emotional and behavioral problems. At-risk children included the preschoolers who were identified as at risk by any reliable screening tool or nominated by teachers as demonstrating externalizing behavior patterns, including chronic problem behaviors, aggressive behaviors, oppositional–defiant behaviors, frequent tantrums, noncompliance, and rule infractions. Studies were excluded if they mainly included participants identified as with autism spectrum disorder or developmental disabilities.
3. Design: Studies had to use group research designs, including an experimental design, quasi-experimental design, or partial randomized control trials with randomization among classrooms or schools. This criterion was also set to ensure the quality of the included studies was high. Studies using single-case research, descriptive studies, qualitative research, or literature reviews were excluded.
4. Independent variables: The social skills interventions could be based on any of the following theoretical frameworks, including behavioral, cognitive–behavioral interventions, etc. Studies focused on inclusion policy or studies on environment rearrangement were excluded.
5. Implementer: Intervention implementers had to be teachers, para-professionals, or therapists who provided services in preschool settings. Studies on parent training or interventions aimed to improve parent–child relationships and interactions were excluded.
6. Settings: Interventions conducted in Head Start programs, public or private preschool programs, daycare programs, or state-funded prekindergarten programs. Because the authors intended to find some commonly applicable group social skills interventions that can be applied in any preschool setting to benefit preschoolers with diverse backgrounds. Studies that were conducted in clinic settings or psychiatric hospitals were excluded.
7. Dependent variables: The interventions had to target one of three skills covered in Gresham’s framework of social skills training: social interactions, prosocial behaviors, or social–cognitive skills. Studies had to examine and report the effects of SSIs on one or more social, emotional, or behavioral outcomes.
8. Results: Studies had to report enough statistics to calculate an effect size for the intervention. Enough statistics means the authors need to report the mean scores and standard deviations of the experiment and control groups that could lead to the calculation of effect size *d* or the authors reported the numbers of participants in the experiment and control groups and any summary effect size statistics (e.g., *t*, *F*, or chi-square) that can be converted to effect size *d*. Studies that did not report enough statistical information to calculate the effect sizes were excluded. For several studies, the authors were contacted to obtain more information to calculate effect sizes. The studies in which the author replied with enough information were included (e.g., [23,24]).

**Reliability for screening.** The titles and full text of the articles were screened by a primary rater. A second rater was trained to reach 90% reliability standard, and she independently screened 25% of all the full-text screening articles using the criteria described above. When the primary rater and the secondary rater made the same decision regarding whether to include one article, we counted it as agreement, and when the primary rater and the secondary rater made different decisions regarding the same article, we counted it as disagreement. Interrater reliability was calculated using the agreements between the primary rater and the second rater divided by the total of agreements and disagreements between the two raters. The reliability between the second rater and the first author was 95%. The raters had meetings to go over the articles with which they had to resolve disagreements.

### 2.3. Coding for Descriptive Information

Included articles were reviewed, and descriptive information was extracted. Each article was coded for (a) participants’ characteristics, (b) methodological features, (c) intervention features, (d) potential moderators of outcomes, and (e) outcome variables.

**Participant characteristics**. Participants were coded on (a) age, (b) gender, (c) ethnicity, (d) the number of all the participants and the number of children in experimental and control group, (e) socio-economic status, and (f) educational setting. The age of the participants was coded as months. Gender was dichotomous as male and female. Ethnicity included Caucasian, African American, Hispanic, Asian, and not reported. For the number of participants, we added the number of children in experimental and control groups if there were only two groups in the study. Several studies included three groups; we used the group with more treatment elements or enhanced treatment as the experimental group and the group with no treatment as the control group [24,25,26,27,28,29]. The data for the third group were not used in the current review. Educational setting was coded as Head Start, preschool/child care setting, special education classroom, or combination of general education and special education classrooms.

**Methodological features.** Research design and randomization were coded for each study. Research design was coded as either experimental design, quasi-experimental design, or partial randomized trial (randomized by classroom or school). Randomization was dichotomous, as either randomized or not. Studies were rated as randomized if they did randomization for individual children, classrooms, or schools.

**Intervention features.** Intervention features included (a) implementer, (b) intervention tier, (c) integration, (d) duration, (e) literacy component, (f) curriculum, (g) parent training component, (h) coaching, and (i) treatment fidelity. Implementer was coded as *researcher*, *trained teacher* or *staff*, and *collaboration among researcher*, *coach and teachers*. Intervention tier was coded as *studies only include primary intervention*, *secondary* or *tertiary intervention,* or *studies include combined intervention tiers*. Integration was coded as *integrated full-day* or *implemented in scheduled time*. *Integrated full-day* referred to the interventions that included strategies that could be implemented throughout the day in preschool settings. *Implemented in scheduled time* referred to interventions in which the sessions were delivered in a scheduled time that fit into the school day. Duration was coded dichotomously as *equal or shorter than 8 weeks* or *longer than 8 weeks*. Literacy component, curriculum, parent training, and coaching were coded dichotomously, as either included in the intervention or not. Interventions that included a literacy component were the programs with elements related to language learning or phonemic awareness. Interventions that did not include a literacy component mainly focused on social skills or social–emotional skills intervention. Interventions with curriculum were those interventions taught according to a certain curriculum. Parent training referred to interventions that included parent training or take-home materials provided along with the interventions implemented in classrooms. Coaching referred to whether any kind of consultation, feedback, or meeting was provided to the implementers along with the implementation of the interventions besides professional development training. Treatment fidelity was coded as reported or not.

**Potential moderators.** Curriculum, intervention tier, integration, randomization, and treatment fidelity were examined as potential moderators. They were examined to determine whether they could explain the differences in outcome measures among different interventions.

**Outcome variables.** All the outcomes related to social–cognitive skills, problem behaviors, and social competence were obtained from each included study. Social–cognitive skills referred to any specific skills related to social–emotional development, such as emotion recognition, emotion regulation skills, and anger control. Problem behaviors included aggressive behaviors, disruptive behaviors, and disengaged/off-task behaviors. Social competence referred to broad construct as an overall evaluation of children’s social competence, such as social competence skills [30] and the preschool competence questionnaire (PCQ) [31].

### 2.4. Inter-Rater Reliability for Coding

The primary rater completed all the coding for the included articles; the second rater received reliability training and independently coded 28% of all the included articles. Interrater reliability was calculated using the agreements divided by the total of agreements and disagreements. The reliability was 93%. The raters had meetings to go over the articles on which they had disagreements in order to resolve those disagreements.

### 2.5. Data Analysis and Calculation of Effects

Hedge’s g [32] was chosen as the effect size statistic for this study as some of the included studies were not randomized group designs, and Hedge’s g can adjust for the pre-intervention differences between the experiment and control group in the calculation process (e.g., [33]). All effect sizes were calculated such that positive values suggested a favorable result for the intervention group over the control group. For most included studies, the group mean differences between the experiment group and control group on the posttest scores adjusted with the pretest scores were used to calculate the effect size. For the studies including three groups, the same rules mentioned above were followed. Most effect sizes were calculated from means and standard deviations or raw data reported in the study. When this information was unavailable, effect sizes were estimated from other statistics (e.g., *t*, *F*, or chi-square).

Robust variance estimation in meta-regression analysis is a method used for addressing statistical dependency among effect sizes [34,35]. We used robust variance estimation meta-analysis to calculate the mean effect size of all the effect sizes obtained from the included studies. The measures reported included teacher-rating scales, parent-rating scales, children taking tests, and results from direct observation. Mean effect sizes for three different outcomes (social–cognitive skills, problem behaviors, and social competence) were calculated.

The significance of the heterogeneity of a group of ESs was examined through the *I*-square statistics. The *I*^2^ statistic represents the percentage of total variance that was caused by heterogeneity rather than chance [36]. According to the benchmark proposed by Higgins et al. [36], an *I*^2^ over 75% indicates high heterogeneity across included studies.

## 3. Results

A total of 1921 articles were identified with the above search strategies. After screening the title and abstracts, 1711 articles were excluded in the first round because they did not meet the inclusion criteria. Next, 210 articles were included for full-text screening. After full-text screening, 25 articles met the inclusion criteria and were included in the review (see Figure 1). Twenty-five articles with a total of 3484 preschoolers met the inclusion criteria. The sample used in the study by Feil et al. [37] was a subgroup of the sample in Feil et al. [1].

The main descriptive features of the qualifying studies are summarized in Table 1. Among the 25 studies, 10 used an experimental design, 6 used a quasi-experimental design, and 9 used a partial randomized design. As to settings, 6 studies were conducted in the Head Start program, 15 were conducted in a public or private preschool classroom, 1 study was conducted in an inclusive preschool classroom with children identified with special needs, and 3 studies were conducted in Head Start classrooms and public preschool classrooms.

Regarding intervention features (see Table 2), interventions were delivered according to certain curricula in 16 studies, while the other 9 did not use any curriculum. A total of 6 studies delivered the SSIs along with an emergent literacy component (e.g., reading or phonemic awareness), whereas 19 other studies did not include a literacy component. Considering multi-tiered systems of support, 10 included studies had a primary intervention, 7 studies had a secondary intervention, 1 study had a tertiary intervention, and the other 7 studies had combined interventions of primary and secondary interventions or three intervention tiers. A total of 14 studies included strategies that could be integrated over a full day to help with children’s social behaviors, and 11 studies only delivered interventions at scheduled time periods. Besides 1 study that did not report the duration of intervention, 7 included studies have interventions lasting for less than or equal to 8 weeks; 17 studies had interventions lasting for more than 8 weeks. A total of 11 studies included parent training or take-home materials for parents; 14 studies did not provide any parent training or materials. A total of 13 studies included coaching to provide feedback or consultation for implementers to adjust their instruction in their classrooms; 12 studies did not provide any coaching other than professional development training at the beginning of the studies. Fifteen studies were implemented by trained teachers or staff, two studies were implemented by researchers, and eight were implemented by researchers and teachers. Sixteen studies reported fidelity, while nine did not report.

### 3.1. Overall Effects

Outcome variables related to social behaviors were obtained from each included study. The measures reported included teacher-rating scales, parent-rating scales, children taking tests, and results reported by other observers. In all, 151 effect sizes were obtained from the 25 included articles in total. Most of the articles reported more than one measurement. Considering the independence among the effect sizes reported in one study, robust variance estimation was used to calculate the mean effect size, and the results were presented in Table 3. The mean effect size was 0.54 (95% CI = 0.42–0.66), indicating a small-to-moderate effect of SSIs on preschoolers’ behavioral outcomes. One mean effect size for all teacher-rating scales, parent-rating scales, children taking tests, and other observers was calculated. The magnitude for these means was the teacher-rating scale average of 0.48 (95% CI = 0.35–0.62), parent-rating scale average of 0.26 (95% CI = 0.04–0.48), children taking tests with an average of 0.69 (95% CI = 0.43–0.94), and the other observer reports average was 0.51 (95% CI = 0.16–0.86). All the mean effect sizes indicated a medium-to-large effect size except for the parent rating scale average.

The average effect sizes for each outcome variable, including social–cognitive skills, problem behaviors, and social competence, were obtained to examine whether SSIs had differential effects on different outcome variables (see Table 3). The average effect sizes for social–cognitive skills, problem behaviors, and social competence were 0.54 (95% CI = 0.42–0.66, *n* = 23), 0.55 (95% CI = 0.39–0.70, *n* = 19), and 0.20 (95% CI = −0.01–0.41, *n* = 4), respectively. Thus, SSIs had a bigger effect on social–cognitive skills and problem behaviors than social competence.

### 3.2. Examining for Publication Bias

**Figure 2:**
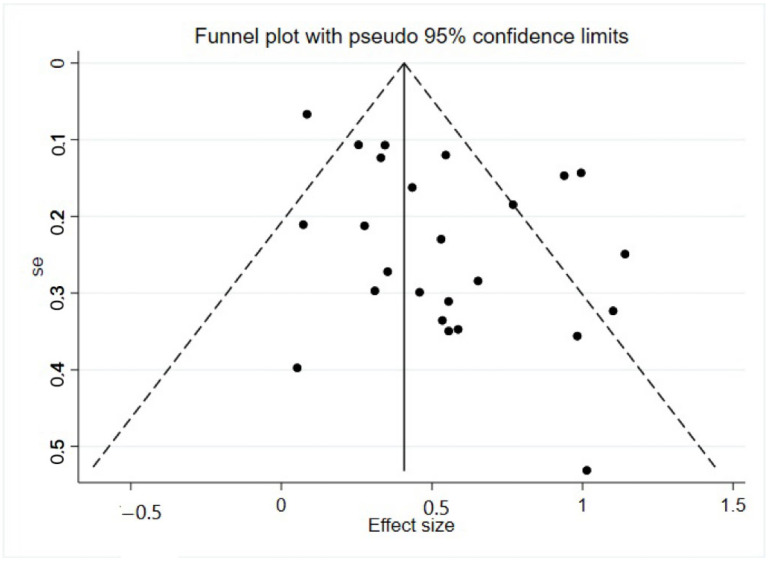
Funnel Plot with Pseudo 95% Confidence Limits.

A funnel plot was created to check the presence of publication bias. In the funnel plot, the standard error was plotted on the y-axis, and the effect size was plotted on the x-axis. We worked with the assumption that if publication bias existed, the funnel would be asymmetric. As shown in Figure 2, some studies with null or negative findings may not have been identified in our search process.

### 3.3. Moderator Analysis

Heterogeneity was examined by sub-group analysis. Five substantive and methodological features of the studies were used to model variations in outcomes: curriculum, intervention tier, integration, randomization, and treatment fidelity. A meta-analysis regression model was used in the R program to analyze these potential moderators (see Table 4).

Curriculum. Studies with a developed curriculum (*n* = 16) were compared to those not using a curriculum (*n* = 9). The *I*^2^ dropped to 69.86, which means that the use of a curriculum explained some of the variance among the studies. The coefficient for the curriculum was −0.30 (*p* < 0.05), indicating that the effect size for interventions delivered using a curriculum was smaller than the interventions without a curriculum.

Integration. Studies with social–behavioral strategies to improve or reinforce children’s social skills that can be integrated throughout the day (*n* = 14) were compared to those that only delivered intervention during scheduled times (*n* = 11). The *I^2^* dropped to 68.88, meaning integration explained some of the variance among the studies. The coefficient for integration was −0.29 (*p* < 0.05), indicating the effect size for the interventions with a full integrated day was significantly smaller than the interventions delivered during scheduled times.

Treatment fidelity. We coded “1” for studies that reported treatment fidelity (*n* = 16) and “0” for studies that did not report treatment fidelity (*n* = 9). The *I*^2^ dropped to 69.22, meaning treatment fidelity explained some of the variance among the studies. The coefficient for treatment fidelity was −0.28 (*p* < 0.05), indicating the effect sizes for the studies that reported treatment fidelity were smaller than the studies that did not report treatment fidelity. The result was the opposite of our hypothesis.

Randomization. We coded “1” for the studies with randomization either by individual, class, or school (*n* = 21) and “0” for studies without randomization (*n* = 4). The coefficient for randomization was 0.26 (*p* < 0.10), meaning when the samples were randomized, the effect size increased compared to studies without randomization. However, the differences between studies with randomization and studies without randomization were not statistically significant.

Intervention tier. We coded “1” for studies that involved primary intervention (*n* = 17) and “0” for secondary or tertiary interventions that included a certain level of individualization (*n* = 8). The coefficient for the intervention tier was -.02, which was not statistically significant.

## 4. Discussions

This meta-analysis summarized the effects of SSIs on preschoolers with or at risk of emotional and behavioral problems. The current review is the first meta-analytic review on SSIs focused specifically on preschoolers with or at risk of emotional and behavioral problems. The descriptive features of the included studies were presented in Table 1, and the features of the study design and participant characteristics were presented in Table 2. The first research question focused on examining the overall effect across studies. The average effect size across all the 25 included studies (*M* = 0.54) indicated moderate effects of SSIs on children’s social behaviors for preschoolers at risk of emotional and behavioral problems. This overall finding is consistent with previous research, which has found small-to-moderate effects for students with EBD across age groups [10,20,21,22]. The second research question focused on the differential effects of SSIs in three areas: (a) social–cognitive skills, (b) problem behaviors, and (c) social competence. The effects of SSIs on social–cognitive skills (*M* = 0.54) and problem behaviors (*M* = 0.54) were comparable and larger than the effects for social competence (*M* = 0.20). This finding is consistent with previous research wherein SSIs had larger effects on specific skill outcomes targeted for intervention than the overall construct of social competence. For example, Beelmann, Pfingsten, and Lösel [21] found larger effect sizes on specific social skills than on the broader construct of social adjustment. Social competence is a broader construct that refers to the overall judgment of a child’s social functioning [13]. The SSIs included in this review had moderate overall effect sizes in improving social–cognitive skills and decreasing problem behaviors than on social competence, indicating that transfer of training and providing an intervention large enough to impact a global evaluation of social competence is still an issue perplexing the field.

The third research question was to explore whether there were any differential effects for SSIs based on the potential moderating variable of curriculum, intervention tier, integration, randomization, and treatment fidelity. Given the heterogeneity of the effect sizes for the included studies, a meta-regression analysis was conducted to examine the factors that accounted for the variabilities. Curriculum, intervention tier, integration, randomization, and treatment fidelity were examined as potential moderators. Of these, three moderating variables (curriculum, integration, and treatment fidelity) were found to be significant. The effect size for the intervention delivered using curriculum-based interventions was smaller than the interventions without curriculum. Therefore, it is not necessary for SSIs to be implemented with a formal curriculum in order to be effective in improving preschoolers’ social behaviors. The effect sizes for interventions with strategies that can be integrated full-day were smaller than the intervention only delivered during the scheduled time. Although an integrated approach to social skills training seems important for the generalization of training effects, the results imply SSIs delivered at scheduled times can be even more effective for preschoolers. The effect size for studies that reported treatment fidelity was smaller than those studies that did not report treatment fidelity. Sixty-four percent of all the included studies reported treatment fidelity. Five articles out of the nine articles that did not report treatment fidelity were published before 2000. This finding is consistent with the findings in a recent review conducted by Wheeler, Mayton, Ton, and Reese [51] that focused on evaluating treatment fidelity across studies aimed at social and emotional skill development. There are possible extraneous factors, such as the publishing year, impacting the effect size.

Studies with randomization were not found to be significant when compared to those without randomization. Twenty-one included studies randomized the participants into an experimental group and a control group. Some of the studies were randomized by individual [25,40] while some of the studies were randomized by classroom [1,38] or school [24]. Only four included studies were not randomized. Although there is no significant difference in the effect sizes for studies with randomization and those without randomization, it is still recommended to randomize participants into different groups to ensure the internal validity of the studies. No significant differences among studies using different tier interventions were found either. Only one of the included studies focused on intensive tertiary interventions, which also points to the need for further research to develop more intensive social skill intervention strategies.

### Limitations and Future Research

In the literature search process, only published articles were included in the review. Dissertations and conference reports were not included. There are different opinions in the field of meta-analysis [52] regarding whether to include studies outside the peer-reviewed literature. Cochrane group [53] recommended using the peer-review process as an initial screening criterion to develop a pool of studies. However, some methodologists in meta-analysis have argued that reviews should include unpublished literature in order to cast a wider net, including studies that may not be published due to a lack of effects [52]. In this review, we focused on the published peer-reviewed literature to ensure the quality of the included articles was good. However, we cannot discount that if some unpublished studies were included, the results may be different. Second, we acknowledge the limitation in the time range of the literature search; this review only included articles published before 2017 as this review is part of a larger review project, and we included some articles published more recently in another review. Third, we used robust variance estimation in order to take the dependency among effect sizes in the included studies. However, robust variance estimation is often limited to the reviews wherein the number of included studies is large, although it may be used to study numbers of as few as 10 [54]. Fourth, there is big heterogeneity among the preschool programs in the included articles. Some people may raise concerns that the differences in the program characteristics and curriculum may impact the results differently. But, we intended to find some social skills instruction that is applicable and effective across different programs so that children with diverse backgrounds could benefit from the instruction. Finally, some included studies included preschoolers and primary school children. These studies (e.g., [23,24]) did not report their results separately but met the inclusion criteria of including preschool students.

Future research is needed to more fully develop SSIs for young children. Learning social skills is a complicated process for young children, especially those with risk factors that are associated with emotional and behavioral problems. Thus, these students may take a longer time to acquire and become fluent in skills as well as transfer those skills to novel settings. Gresham et al. [55] reported that the typical SSI averaged 2.5–3.0 h per week for 10–12 weeks in their review and speculated the total time for SSIs may be insufficient to remediate social skills deficits and that SSIs for longer durations may need to be implemented.

More studies that focus on tertiary-tier SSIs are needed. Only one study focused on a tertiary-tier level intervention among the included studies in this review. Gresham [14] also called for future research to develop more intensive SSI strategies for students with or at risk of emotional and behavioral problems. In addition, studies focusing on internalizing problem behaviors are needed. Only one study focused on children with internalizing problem behaviors (i.e., [26]), and one study included children with aggressive behaviors or withdrawn behaviors (i.e., [42]) in the current review. Although internalizing problem behaviors are usually not disruptive in the classroom, internalizing problems such as social withdrawal show the greatest stability across developmental periods [56]. With the findings from this review, SSIs continue to be a promising but, as Gresham has indicated, largely unfulfilled approach to improving social behaviors for preschoolers at risk of emotional and behavioral problems [55].

## Figures and Tables

**Table 1:** Descriptive features of the included articles.

Methodological Features	N	%	Intervention Features	N	%	Intervention Features	N	%
Research design			Intervention tier			Setting		
Experimental design	10	40	Primary intervention	10	40	Head Start	6	24
Quasi-experimental design	6	24	Secondary intervention	7	28	Public or private preschool	15	60
Partial randomized	9	36	Tertiary intervention	1	4	Inclusive preschool	1	4
Randomization			Combined	7	28	Combination of the above	3	12
Yes	21	84	Integration of intervention			Literacy component		
No	4	16	Integrated full-day	14	56	Yes	6	24
Curriculum			Scheduled time period	11	44	No	19	76
Yes	16	64	Implementer			Coaching		
No	9	36	Researcher	2	8	Yes	13	52
			Trained teacher or staff	15	60	No	12	48
			Collaboration	8	32			

**Table 2:** Study design and participants characteristics for the included studies.

ID	Study	Design	N	n Exp	n Con	Gender	Ethnicity	Disability	Intervention	Duration	Tier	Parent	Coach	T.F.
1.	Bierman et al., 2008 [18]	Exp.	356	192	164	54% F	17% H, 25% AA	At risk, low income	The Head Start REDI program and PATHS Curriculum	33 wks	1st	2	Y	Y
2.	Celik et al., 2016 [38]	Exp.	22	11	11	9% F	NR	At risk of antisocial	preschool FSS	30 days	3rd	1	Y	Y
3.	Denham et al., 1996 [39]	Quasi	105	63	42	NR	76% min	69 at risk	High Scope model, PATHS, Prosocial Activity Guide, and ICPS	32 wks	1st + 2nd	3	Y	Y
4.	Feil et al., 2014 [1]	Exp.	126	65	61	35% F	31% AA, 44% C, 13% H	Ext. problems	preschool FSS	30 days	2nd	1	Y	Y
5.	Feil et al., 2016 [37]	Exp.	45	26	19	16% F	16% AA,	12% have disability	preschool FSS	30 days	1st + 2nd	1	Y	Y
6.	Fishbein et al., 2016 [24]	Exp.	327	154	173	NR	Most AA	NR	PATHS, universal social–emotional program	22 wks	1st	2	Y	Y
7.	Graziano et al., 2016 [25]	Exp.	41~	15	11	24% F	84% H	Ext. problems	School readiness parenting program and STP-PreK	8 wks	2nd	1	N	Y
8.	Gunter et al., 2012 [26]	Quasi	84~	28	32	50% F	66.7% H, 26.2% C, 2.4% AA,	Int. problems	Strong Start Pre-K	6 wks	1st	3	N	Y
9.	Hart et al., 2016 [40]	Exp.	46 *	22	24	22% F	98% min, 52% H	Ext. problems	Kindergarten summer readiness classroom	4 wks	2nd	1	Y	Y
10.	Hemmeter et al., 2016 [41]	Exp.	97 *	54	43	NR	42.3% C, 37.5% AA, 18.3% H	72% with IEP	Pyramid Model for Promoting Young Children’s Social–Emotional Competence	1 school year	1st + 2nd + 3rd	3	Y	Y
11.	Hughes et al., 2015 [27]	Quasi	57~	20	17	50.8% F	NR	NR	PATHS	9 months	1st	3	N	N
12.	Hyatt, 2007 [28]	Exp.	24~	8	8	50% F	NR	6 have disability	Adaptation from Skill streaming in Early Childhood	8 days	2nd	3	N	N
13.	Koglin et al., 2011 [42]	Quasi	90	48	42	47.4% F	NR	Ext. or Int. behavior	Behavior training for preschool children	13 wks	1st	3	N	Y
14.	Nix et al., 2013 [43]	Exp.	356	192	164	54% F	25% AA, 17% L	At risk, low income	REDI and preschool PATHS	1 year	1st	3	Y	Y
15.	Reinke et al., 2014 [44]	Exp.	46	23	23	30% F	91.3% AA, 8% C	15% with disruptive behaviors	IY Teacher Classroom Management,	NR	1st + 2nd	3	Y	Y
16.	Serna et al., 2000 [45]	Exp.	84	53	31	56% F	71.4% H, 12% AA	NR	Self-determination program	12 wks	1st	1	N	N
17.	Sharp, 1981 [29]	Exp.	54~	18	19	46% F	All AA	NR	ICPS	11 wks	2nd	3	N	N
18.	Shure et al., 1980 [17]	Exp.	219	113	106	55.7% F	All AA	NR	ICPS	3 months	2nd	3	N	N
19.	Shure et al., 1972 [30]	Exp.	54~	22	21	48% F	NR	NR	ICPS	10 wks	2nd	3	N	N
20.	Stefan, 2008 [46]	Quasi	52	26	26	50% F	NR	NR	A 5-step social–emotional program	5 months	1st	3	N	N
21.	Stefan et al., 2013 [47]	Exp.	158	89	69	55% F	All C	20% Ext. problem	Social–emotional prevention program	NR	1st + 2nd	1	Y	Y
22.	Tankersley et al., 1996 [48]	Exp.	45	34	11	37.8% F	64% AA, 33% C, 2% H	At risk, with Ext. problems	Affection activities, social skills instructions,	10 wks	2nd	1	N	N
23	Tucker et al., 2017 [49]	Exp.	206	107	99	NR	40% AA, 22%NA, 20% H	9 have disability	Sunshine circles (group therapy)	1 year	1st	3	Y	N
24.	Webster-Stratton et al., 2008 [23]	Exp.	1768	1096	672	50% F	18% L, 18% AA, 20% A, 27% C	At risk, low income	IY social–emotional and problem-solving curriculum	1 year	1st + 2nd	3	Y	Y
25.	Xu, 2015 [50]	Exp.	75	39	36	52% F	83% H	NR	Adapted peer tutoring	1 semester	1st	3	N	Y

Notes: Exp. = experimental design; Quasi = Quasi-experimental design; F = female; NR = not reported; H = Hispanic; AA = African American; C = Caucasian; min = minority; L = Latino; NA = North African; Ext. = externalizing; Int. = internalizing; REDI = Research based, developmentally informed; PATHS = Promoting Alternative Thinking Strategies; FSS = First Step to Success; ICPS = Interpersonal Cognitive Problem Solving; STP-PreK = summer treatment program for prekindergarteners; IY = incredible years; wks = weeks; T.F. = treatment fidelity. * the number of students analyzed in the results; ~ the total number of three groups in the study.

**Table 3:** Robust variance estimate effect sizes by outcome and observer.

		Outcome			
		Social–Cognitive Skills	Problem Behavior	Social Competence	Effect Size
**Respondent**	**Teacher**	0.51 ^1^ (0.35, 0.67) 2.00 (24:12)	0.53 ^1^ (0.38, 0.69) 3.18 (54:17)	0.23 ^2^ (0.03, 0.42) 1.50 (6:4)	0.48 ^1^ (0.35, 0.62) 4.67 (84:18)
	**Parent**	0.37 ^2^ (0.05, 0.70) 1.25 (5:4)	0.24 (−0.03, 0.51) 1.71 (12:7)	0.08 (−0.41, 0.56) 1.50 (3:2)	0.26 ^2^ (0.04, 0.48) 2.86 (20:7)
	**Child**	0.69 (0.43, 0.94) 3.00 (24:8)	no effect size estimates	no effect size estimates	0.69 ^1^ (0.43, 0.94) 3.00 (24:8)
	**Other**	0.63 ^2^ (0.13, 1.13) 2.50 (15:6)	0.58 (−0.24, 1.40) 1.75 (7:4)	0.26 (0.05 to 0.47) *(single effect size)*	0.51 ^2^ (0.16, 0.86) 2.88 (23:8)
**Overall Summary**		0.54 ^1^ (0.42, 0.66) 2.96 (68:23)	0.54 ^1^ (0.39, 0.70) 3.84 (73:19)	0.20 (−0.01, 0.41) 2.50 (10:4)	0.54 ^1^ (0.42, 0.67) 6.04 (151:25)
***Legend***			Effect Size (95% Confidence Interval) Average **number of** Effect Size per Study (number of Effect Size: number of Studies).		

Notes. ^1^ Statistically significant, α = 0.01; ^2^ Statistically significant, α = 0.05; The statistics in each cell were listed following this format.

**Table 4:** Meta-regression analysis results.

Meta-Regression Models	Coeff.	*p*-Value	95% CI	*I*^2^ tau^2^
Intercept	0.75	<0.01	0.48, 1.02	69.86
Curriculum	−0.30	<0.05	−0.58, −0.03	0.0923
Intercept	0.72	<0.01	0.51, 0.93	68.88
Integration	−0.29	<0.05	−0.52, −0.06	0.0877
Intercept	0.74	<0.01	0.56, 0.92	69.22
Treatment fidelity	−0.28	<0.05	−0.50, −0.06	0.0891
Intercept	0.33	<0.10 *	−0.00, 0.65	73.46
Randomization	0.26	<0.10	−0.07, 0.59	0.1067
Intercept	0.55	<0.01	0.39 0.72	74.23
Intervention tier	−0.02	ns	−0.27, 0.22	0.1154

Notes: * df less than 4.

## Data Availability

Data available upon request.

## References

[1] Feil E.G., Frey A., Walker H.M., Small J.W., Seeley J.R., Golly A., Forness S.R. (2014). The efficacy of a home-school intervention for preschoolers with challenging behaviors: A randomized controlled trial of Preschool First Step to Success. J. Early Interv..

[2] Brown W.H., Conroy M.A. (2011). Social-emotional competence in young children with developmental delays: Our reflection and vision for the future. J. Early Interv..

[3] Forness S.R., Freeman S.F., Paparella T., Kauffman J.M., Walker H.M. (2012). Special education implications of point and cumulative prevalence for children with emotional or behavioral disorders. J. Emot. Behav. Disord..

[4] Kellam S.G., Werthamer-Larsson L., Dolan L.J., Brown C.H., Mayer L.S., Rebok G.W., Anthony J.C., Laudolff J., Edelsohn G., Wheeler L. (1991). Developmental epidemiologically based preventive trials: Baseline modeling of early target behaviors and depressive symptoms. Am. J. Community Psychol..

[5] McCabe P.C., Altamura M. (2011). Empirically valid strategies to improve social and emotional competence of preschool children. Psychol. Sch..

[6] Maag J.W. (2006). Social Skills Training for Students With Emotional and Behavioral Disorders: A Review of Reviews. Behav. Disord..

[7] Bulotsky-Shearer R.J., Domínguez X., Bell E.R., Rouse H.L., Fantuzzo J.W. (2010). Relations between behavior problems in classroom social and learning situations and peer social competence in Head Start and kindergarten. J. Emot. Behav. Disord..

[8] Heckman J.J. (2006). Skill formation and the economics of investing in disadvantaged children. Science.

[9] Gresham F.M. (2007). Response to intervention and emotional and behavioral disorders: Best practices in assessment for intervention. Assess. Eff. Interv..

[10] Lösel F., Beelmann A. (2003). Effects of Child Skills Training in Preventing Antisocial Behavior: A Systematic Review of Randomized Evaluations. Ann. Am. Acad. Political Soc. Sci..

[11] Sentse M., Kretschmer T., de Haan A., Prinzie P. (2017). Conduct problem trajectories between age 4 and 17 and their association with behavioral adjustment in emerging adulthood. J. Youth Adolesc..

[12] Merrell K.W., Gimple G. (2014). Social Skills of Children and Adolescents: Conceptualization, Assessment, Treatment.

[13] Gresham F.M. (1986). Conceptual issues in the assessment of social competence. Children’s Social Behavior: Development, Assessment, and Modification.

[14] Gresham F. (2015). Evidence-based social skills interventions for students at risk for EBD. Remedial Spec. Educ..

[15] Gresham F.M., Cook C.R., Crews S.D., Kern L. (2004). Social skills training for children and youth with emotional and behavioral disorders: Validity considerations and future directions. Behav. Disord..

[16] Krueger R.F., Hicks B.M., McGue M. (2001). Altruism and Antisocial Behavior: Independent Tendencies, Unique Personality Correlates, Distinct Etiologies. Psychol. Sci..

[17] Shure M.B., Spivack G. (1980). Interpersonal problem solving as a mediator of behavioral adjustment in preschool and kindergarten children. J. Appl. Dev. Psychol..

[18] Bierman K.L., Domitrovich C.E., Nix R.L., Gest S.D., Welsh J.A., Greenberg M.T., Blair C., Nelson K.E., Gill S. (2008). Promoting academic and social-emotional school readiness: The Head Start REDI program. Child Dev..

[19] Kauffman J.M., Clough P. (2005). How we prevent the prevention of emotional and behavioral disorders. Handbook of Emotional and Behavioural Difficulties.

[20] Schneider B.H., Byrne B.M. (1985). Children’s social skills training: A meta-analysis. Children’s Peer Relations: Issues in Assessment and Intervention.

[21] Beelmann A., Pfingsten U., Lösel F. (1994). Effects of training social competence in children: A meta-analysis of recent evaluation studies. J. Clin. Child Psychol..

[22] Schneider B.H. (1992). Didactic methods for enhancing children’s peer relations: A quantitative review. Clin. Psychol Rev..

[23] Webster-Stratton C., Jamila Reid M., Stoolmiller M. (2008). Preventing conduct problems and improving school readiness: Evaluation of the incredible years teacher and child training programs in high-risk schools. J. Child Psychol. Psychiatry.

[24] Fishbein D.H., Domitrovich C., Williams J., Gitukui S., Guthrie C., Shapiro D., Greenberg M. (2016). Short-term intervention effects of the PATHS curriculum in young low-income children: Capitalizing on plasticity. J. Prim. Prev..

[25] Graziano Paulo A., Hart K. (2016). Beyond behavior modification: Benefits of social-emotional/self-regulation training for preschoolers with behavior problems. J. Sch. Psychol..

[26] Gunter L., Caldarella P., Korth B.B., Young K.R. (2012). Promoting social and emotional learning in preschool students: A study of Strong Start Pre-K. Early Child. Educ. J..

[27] Hughes C., Cline T. (2015). An evaluation of the preschool PATHS curriculum on the development of preschool children. Educ. Psychol. Pract..

[28] Hyatt K.J., Filler J.W. (2007). A comparison of the effects of two social skill training approaches on teacher and child behavior. J. Res. Child. Educ..

[29] Sharp K.C. (1981). Impact of interpersonal problem-solving training on preschoolers’ social competency. J. Appl. Dev. Psychol..

[30] Shure M.B., Spivack G., Gordon R. (1972). Problem-solving thinking: A preventive mental health program for preschool children. Lit. Res. Instr..

[31] Olson S.L. (1989). Assessment of impulsivity in preschoolers: Cross-measure convergences, longitudinal stability, and relevance to social competence. J Clin Child Psychol.

[32] Hedges L.V., Olkin I. (2014). Statistical Methods for Meta-Analysis.

[33] Wilson S.J., Lipsey M.W. (2007). School-based interventions for aggressive and disruptive behavior: Update of a meta-analysis. Am. J. Prev. Med..

[34] Hedges L.V., Tipton E., Johnson M.C. (2010). Robust variance estimation in meta-regression with dependent effect size estimates. Res. Synth. Methods.

[35] Tanner-Smith E.E., Tipton E. (2014). Robust variance estimation with dependent effect sizes: Practical considerations including a software tutorial in Stata and SPSS. Res. Synth. Methods.

[36] Higgins J.P., Thompson S.G., Deeks J.J., Altman D.G. (2003). Measuring inconsistency in meta-analyses. BMJ.

[37] Feil E.G., Small J.W., Seeley J.R., Walker H.M., Golly A., Frey A., Forness S.R. (2016). Early intervention for preschoolers at risk for attention-deficit/hyperactivity disorder: Preschool first step to success. Behav. Disord..

[38] Çelik S., Diken I., Çolak A., Arikan A., Aksoy F., Tomris G. (2016). Effectiveness of the preschool version of the First Step to Success early intervention program for preventing antisocial behaviors. Educ. Sci.-Theory Pract..

[39] Denham S.A., Burton R. (1996). A social-emotional intervention for at-risk 4-year-olds. J. Sch. Psychol..

[40] Hart K.C., Graziano P.A., Kent K.M., Kuriyan A., Garcia A., Rodriguez M., Pelham W.E. (2016). Early intervention for children with behavior problems in summer settings: Results from a pilot evaluation in head start preschools. J. Early Interv..

[41] Hemmeter M.L., Snyder P.A., Fox L., Algina J. (2016). Evaluating the implementation of the Pyramid Model for promoting social-emotional competence in early childhood classrooms. Top. Early Child. Spec. Educ..

[42] Koglin U., Petermann F. (2011). The effectiveness of the behavioural training for preschool children. Eur. Early Child. Educ. Res. J..

[43] Nix R.L., Bierman K.L., Domitrovich C.E., Gill S. (2013). Promoting children’s social-emotional skills in preschool can enhance academic and behavioral functioning in kindergarten: Findings from Head Start REDI. Early Educ. Dev..

[44] Reinke W.M., Stormont M., Herman K.C., Wang Z., Newcomer L., King K. (2014). Use of coaching and behavior support planning for students with disruptive behavior within a universal classroom management program. J. Emot. Behav. Disord..

[45] Serna L., Nielsen E., Lambros K., Forness S. (2000). Primary prevention with children at risk for emotional or behavioral disorders: Data on a universal intervention for Head Start classrooms. Behav. Disord..

[46] Stefan C.A. (2008). Short-term efficacy of a primary prevention program for the development of social-emotional competencies in preschool children. Cogn. Brain Behav..

[47] Ştefan C.A., Miclea M. (2013). Effects of a multifocused prevention program on preschool children’s competencies and behavior problems. Psychol. Sch..

[48] Tankersley M., Kamps D., Mancina C., Weidinger D. (1996). Social interventions for Head Start children with behavioral risks: Implementation and outcomes. J. Emot. Behav. Disord..

[49] Tucker C., Schieffer K., Wills T.J., Hull C., Murphy Q. (2017). Enhancing social-emotional skills in at-risk preschool students through Theraplay based groups: The Sunshine Circle Model. Int. J. Play Ther..

[50] Xu Y. (2015). Examining the effects of adapted peer tutoring on social and language skills of young English language learners. Early Child Dev. Care.

[51] Wheeler J.J., Mayton M.R., Ton J., Reese J.E. (2014). Evaluating treatment integrity across interventions aimed at social and emotional skill development in learners with emotional and behaviour disorders. J. Res. Spec. Educ. Needs.

[52] Rosenthal R., DiMatteo M.R. (2001). Meta-analysis: Recent developments in quantitative methods for literature reviews. Annu Rev. Psychol..

[53] Higgins J.P., Green S. (2008). Cochrane Handbook for Systematic Reviews of Interventions.

[54] Fisher Z., Tipton E. (2015). Robumeta: An R-package for robust variance estimation in meta-analysis. arXiv.

[55] Gresham F.M., Van M.B., Cook C.R. (2006). Social skills training for teaching replacement behaviors: Remediating, acquisition deficits in at-risk students. Behav. Disord..

[56] Rubin K.H., Asendorpj J.B. (2014). Social withdrawal, inhibition, and shyness in childhood: Conceptual and definitional issues. Social Withdrawal, Inhibition, and Shyness in Childhood.

